# Patients’ experiences of the transcatheter aortic valve implantation trajectory: A grounded theory study

**DOI:** 10.1002/nop2.124

**Published:** 2018-02-04

**Authors:** Karin Olsson, Ulf Näslund, Johan Nilsson, Åsa Hörnsten

**Affiliations:** ^1^ Cardiology, Heart Centre Department of Public Health and Clinical Medicine Umeå University Umea Sweden; ^2^ Department of Nursing Umeå University Umea Sweden

**Keywords:** aortic stenosis, coping, hope, qualitative study, recovery, supportive nursing, transcatheter aortic valve implantation

## Abstract

**Aim:**

The aim of this study was to explore how patients experienced the recovery process from transcatheter aortic valve implantation.

**Design:**

A qualitative approach where in‐depth interviews were used.

**Method:**

Eleven men and eight women undergoing transcatheter aortic valve implantation were individually interviewed 6 months after transcatheter aortic valve implantation. Grounded theory was used for the analysis.

**Results:**

The analysis generated the core concept “A journey of balancing between life‐struggle and hope” connected to descriptive, bipolar categories. Before transcatheter aortic valve implantation patients not only felt threatened but also experienced hope. The rehabilitation phase was described as demanding or surprisingly simple. At the 6 months follow‐up patients were pleased to return to life, however, still struggling with limitations. To feel hope is essential for transcatheter aortic valve implantation patients’ well‐being, both before and during the recovery process. It is important that healthcare professionals not only support hopeful thinking but also take time to discuss and prepare patients, talk about concerns and build confidence. Individual plans for rehabilitation should be designed.

## 1 INTRODUCTION

Aortic stenosis is the most common valve disease in the Western countries and is increasing due to the ageing population. Untreated severe symptomatic aortic stenosis has a very poor prognosis and the mortality is up to 50% 1 year after onset of symptoms and more than 90% after 5 years (Bonow, Leon, Doshi, & Moat, 2016). Surgical aortic valve replacement is the gold standard and it has good results for survival and for health‐related quality of life even in old age (Olsson, Janfjäll, Orth‐Gomér, Undén, & Rosenqvist, 1996; Vahanian & Iung, 2012). Some patients are denied surgery due to, for instance, previous surgery, porcelain‐aorta, old age, left ventricular dysfunction or comorbidities (Iung, 2008). Transcatheter aortic valve implantation (TAVI), where a biological aortic valve is implanted inside the patient's aortic valve, is a less invasive method and has made it possible to treat patients with higher surgical risks (Osnabrugge et al., 2013; Vahanian & Iung, 2012). Before patients are accepted for TAVI they undergo thorough examinations, functional and cognitive tests. The results are then discussed at a multidisciplinary team conference where risk benefits are weighted and decisions about acceptance or not are made (Kappetein et al., 2013). Different techniques may be applied for TAVI. The most common is the transfemoral technique. Patients in this study were treated with a transapical approach, which means that it is performed by a mini‐thoracotomy and implantation of the valve prosthesis through the left ventricular apex under general anaesthesia. Complications connected to TAVI are varied and related to the type of procedure, patient selection and device used. Stroke, vascular complications and paravalvular aortic regurgitation are the most common complications; but major bleeding, new need for pacemaker, new atrial fibrillation, kidney injury, pleural effusions and endocarditis have also been reported (Cao et al., 2013; McRae & Rodger, 2012).

### 1.1 Background

Several studies performed after TAVI have reported a good effect on survival, health‐related quality of life and heart function. Some results were comparable to those from open surgery (Deutsch et al., 2013; Leon et al., 2016). However, knowledge derived from qualitative studies describing patients’ experiences in relation to TAVI are generally lacking. Lauck, Baumbusch et al. (2016) reported factors influencing the patients′ decision of accepting TAVI: symptom burden; the experienced patient; expectations; healthcare system and informal support; logistical barriers and facilitator; and obligations and responsibilities (Lauck, Baumbusch et al., 2016). Two studies from our research group described experiences from patients planned for TAVI (Olsson, Naslund, Nilsson, & Hornsten, 2016a, 2016b). The studies highlighted how patients struggle to cope with increasing symptoms to manage daily life, to preserve self‐esteem and to process the decision to undergo TAVI. We identified different patterns in decision‐making to accept TAVI; the ambivalent, the obedient and the reconciled. In the literature there is no agreement about the meaning of recovery. Theoretical frameworks that exist are connected to recovery from mental health issues or addiction. However, we define recovery according to English Oxford dictionary as “the action or process of regaining possession or control of something lost and/or a return to a normal state of health, mind or strength”. Berg and coworkers reported patients’ experiences of recovery after pulmonary or aortic valve replacement including TAVI. The overall concept was “suffering weakness” and “struggling to resume normality”. Patients felt weak with a changed body, but after a long recovery process during which they felt fragile, regained vitality and returned to their daily life (Berg, Zwisler, Pedersen, Haase, & Sibilitz, 2013). We have not found any study describing patients’ experiences of the whole TAVI trajectory, which is of interest for this group of physically frail patients who often suffer from comorbidities and need special attention from health professionals. With the intention to improve care for this group of patients, the aim of this study was to explore how patients experienced the recovery process from TAVI.

## 2 METHODS

### 2.1 Qualitative method

We have used Grounded theory (GT) in this study. The purpose of the GT method is to explain what happens in the area being studied and is suitable when the process is in focus (Glaser & Strauss, 1968). Charmaz (2006) highlights that GT ethnography gives priority to a studied phenomenon or process rather than describing a setting by itself. Something to which we adhered. The desire was to show the complexities of particular worlds, views and actions, and to conceptualize the studied process, to understand it in a more abstract way. Priority was to identify patterns and connections rather than to seek causality and linear reasoning (cf. Charmaz, 2006).

### 2.2 Setting and participants

The study was performed at a University clinic in Sweden with experience from 1 year of TAVI at time for inclusion. The hospital serves the northern region comprising four county councils cover more than half of Sweden's area and had a population of 885 000 inhabitants in 2015.

All patients (*n* = 24) treated with TAVI for 1 year were consecutively included in the study. They were informed verbally and in writing about the study and all accepted participation. This report concerns the 6 months follow‐up among those who were still alive, *N* = 19. The participants, all Swedish speaking, were individually interviewed in their home or in their county hospital. Data collection was completed in January 2012. Baseline data of the participants are presented in Table 1. They represented a variety of people, who provided an opportunity to find multiple aspects of phenomena in study and the sample size was sufficient to reach saturation.

**Table 1:** Baseline data of the participants

	Men	Women
Number of participants	11	8
Age, md (min‐max)	80 (65–89)	82 (60–90)
Living alone	7	5
Home assistance	0	2
NYHA class III^a^	4	4
NYHA class IV^a^	7	4
Syncope	2	3
Diabetes	2	2
Previous bypass graft surgery	7	3
Previous stroke	3	2
Peripheral vascular disease	3	4
Chronic obstructive lung disease	5	2

Class III: Marked limitation of physical activity. Comfortable at rest. Less than ordinary activity causes fatigue, palpitation, or dyspnoea.

a Class IV: Unable to carry on any physical activity without discomfort. Symptoms of heart failure at rest. If any physical activity is undertaken, discomfort increases (NYHA, 1994).

### 2.3 Ethical considerations

The study was approved by the Regional Ethics Board in Umeå Reg. No. 2011‐340‐31M. Since interviews could cause emotional reactions we were prepared to offer support, but it was never needed. Instead many patients expressed their gratitude for being listened to.

### 2.4 Data collection

Individual, in‐depth interviews were performed and digitally audio recorded by the first author, a nurse with long experience from cardiac care but who was not directly involved in the care of the patients. The interviews had a narrative approach and started with the broad, open‐ended question: Can you please tell me about your experiences of undergoing TAVI and about your situation today? The participants often started their narratives by describing their situation before TAVI, followed by experiences from hospitalization and then their first time at home. The interviews ended with questions about their current situation and view of the future. Follow‐up questions on emerging patterns were incorporated into interviews with subsequent participants to gain a deeper understanding. The interviews were performed either at the outpatient clinic or in the participants’ homes and lasted for 15–60 min (median 35 min) and were transcribed verbatim. The emerging patterns were documented using memos after the interviews and during the entire analytic process.

### 2.5 Data analysis

The analysis was performed by the first author under the supervision of the last author, also a nurse with long experience of qualitative research. Preliminary as well as final results were thoroughly discussed in the whole research group.

Initially, the interviews were read thoroughly to get a first idea of what the text was all about. The text was then initially coded line by line and labelled with words close to data. In the next step, the focused coding, important codes that made the most analytic sense were sorted out and recoded on a more abstract level. An example from the analysis is presented in Table 2. In the theory building phase, we compared, interpreted and analysed how codes were related and linked to each other and categories were created that gave a theoretical direction to the analysis. When the focus codes and categories were saturated, and no more dimensions and properties emerged, they were compared, and their relations were identified to create a theory (cf. Charmaz, 2006). In this constant comparison analysis, we moved back and forth, using our memos, going back to the interviews, reading the text and discussing results in the research team to ensure credibility.

**Table 2:** Example from the analytic process

Focused codes	Initial codes	Transcript
Feeling week Facing death Being exposed	Difficult time after TAVI No strength Tired Reduced power Almost worse than before Prepared to die before TAVI Close to the end Worried not getting helpWorried to die	I: How was time after TAVI? P: It was difficult, I did not have any strength. I was tired, very tired. I had to go to bed and rest in daytime and I felt that I had reduces power… I: Did you feel worse than before TAVI? P: Yes, almost… but then I was ill too. I had asked the doctor, will I live for Christmas if I do not get surgery? So I was prepared to…that I was close to the end…if I did not get help, so therefore I was very worried. If they could not do this, the only thing that remained was…that worried me. Yes I was worried.

### 2.6 Rigour

Trustworthiness in Grounded theory, according to Charmaz, could be defined as credibility, originality, resonance and usefulness. To ensure credibility, I interviewed many patients with various backgrounds. By systematically comparing interviews and categories, I tried to ensure that there was a logical link in the analysis and that it covered the data. The emerging results were discussed in the research group representing various perspectives including two cardiologists, of which one is a TAVI interventionist. Since TAVI is a new area and few studies on patients’ experiences of TAVI are published, the study offers new insights which strengthen originality. I used quotations from the participants in the presentation of the result to demonstrate the resonance, the links between the larger collective and the individual. The result from the study could be useful in the care of the increasing number of patients treated with TAVI (cf. Charmaz, 2006).

## 3 RESULTS

**Figure 1:**
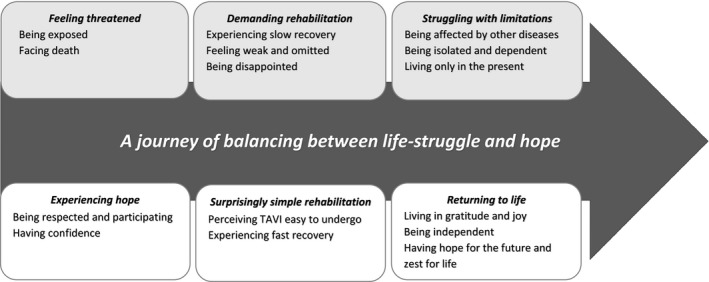
A model of the TAVI trajectory

The analysis of the interviews generated a core concept “A journey of balancing between life‐struggle and hope” connected to descriptive, almost bipolar categories: “feeling threatened” vs. “experiencing hope”; “demanding” vs. “surprisingly simple rehabilitation”; and “struggling with limitations” vs. “returning to life”. Figure 1 provides an illustration of the model and each category is described in the following text. Each quotation is marked with a participant number.

### 3.1 Feeling threatened

The time before TAVI, when participants became weaker and weaker, was described as frightening. This category concerned the threatening situation our patients were facing; to be old and in a bad condition, going through examinations and a new treatment that was life or death.

#### 3.1.1 Being exposed

The time before TAVI was very distressing. Participants had worries about both being denied TAVI and of being accepted and undergoing it. The investigations made before decision of TAVI were also described as difficult to cope with, especially the transoesophageal echocardiography. When risks were discussed when the patient was present but not included, it felt humiliating. The patients knew that TAVI was a new method and some said they felt to some degree like a guinea pig. At the same time, it was described as exciting to be one of the first at the hospital to be offered this procedure. “I understand that they want to try the new method on people and, after all, it's good when everything is going well, but you do not want to feel like a guinea pig either. You have to realize that it must be like that or else there will be no development” 11.

#### 3.1.2 Facing death

Different situations before TAVI were reported, when participants thought they were dying and felt that they became weaker. Men estimated lost strength by relating to how far they could walk in meters or when they last could mow the lawn. Patients had been informed by doctors about the bad prognosis and they had asked questions like “Will I live until Christmas without surgery” 18? Some described how they prepared themselves to die and wondered which way was the best, from the disease or on the operating table.

### 3.2 Experiencing hope

On the other hand, a hopeful feeling for having TAVI was also expressed. Several had previously undergone surgeries or been seriously ill and became healthier, which implied confidence in health care and they were very hopeful about also recovering this time.

#### 3.2.1 Being respected and participating

Having a dialogue with doctors, being informed and participating in treatment decisions, made the patients feel respected. “Me and my doctor, we decided together…” 4. After long discussions, patients were mentally prepared; they knew that TAVI was a new procedure and they were informed of the risks. “We provide no warranty, you must understand, the doctor said. Well, I understand, I said, but you sound hopeful anyway” 18. They had shared their worries with their doctor and did not feel forced to accept TAVI. Having hope was described as vital and occurred in interaction with professionals.

#### 3.2.2 Having confidence

Having confidence in the doctors was very important. When the doctors said that they believed in the TAVI method and that this was the best treatment for them, patients got the courage to go on. “I need to have confidence in the doctors to whom I will entrust myself. After all, I put my life in their hands. It's those things that makes you actually agreeing to this thing [TAVI] that is really vital and there is no guarantee…” 4.

### 3.3 Demanding rehabilitation

This category concerns the problems that appeared for patients during rehabilitation, for example, some had to stay for a long time in hospital after TAVI due to complications or side effects from medication. This was demanding and depressing.

#### 3.3.1 Experiencing slow recovery

Some described slow recovery and tiredness after TAVI that could lead to some time in a rehabilitation clinic. “The expected 3 days at hospital became 3 months.” One of the most serious complications reported was a pulmonary complication which resulted in respiratory therapy for several weeks. Periods of depression, nightmares and even thoughts of suicide during the recovery phase were reported. When having comorbidities, for example, a history of stroke, it could lead to long rehabilitation to be able to walk properly again. Men frequently described urinary problems after catheter removal that could last for a long time. Despite all these problems, patients struggled to get well and to cope with their situation. Those who had undergone coronary bypass surgery in the past and expected recovery to take about the same time were disappointed when it was slower now, but realized that they had grown older and were in worse condition. It was important to maintain hope when the recovery was slow and encouragement from professionals played a major role. “I got good news from the doctor yesterday. He said: it can take a whole year before you get well. So it was a ray of hope, I think. You think you'll be fine at once but it takes time… and that's okay” 9. Moreover, one's own efforts were important to live in hope. Signs of recovery were noted and by exercising and making tests they got proof of improvements. “I have done a test on a small hill. Initially, after surgery, I had to stop and rest, but after a while, I took the hill without resting. Then I was very happy and said to myself: I have really become better” 18.

#### 3.3.2 Feeling weak and omitted

Participants felt weak and tired the first period after TAVI, some more than before. They needed help, but were often suspicious and avoided home care service. Their desire and struggle to cope by themselves was strong and they were unhappy if they became dependent, especially if they had never been dependent before or if relatives did not understand their needs. “The worst thing is to feel so weak and omitted… I am very doubtful that I would do it (TAVI) again, oh, how I've been struggling! Just waking up was hard, I just wanted to sleep. And just to stand up was tough, I fell. I thought I never would be able to walk again. To feel so helpless, yes, I was completely at their mercy… They forced me to get up, it was awful, but it was my rescue” 16.

#### 3.3.3 Being disappointed

Disappointment was expressed when expectations of TAVI did not match the outcome or when complications occurred. One got a stroke about a month after TAVI and her life situation was described as much worse than before. Although the TAVI procedure succeeded, she sometimes regretted that she accepted it. Refraining from finding out facts and risks about the TAVI procedure was a strategy sometimes used to manage fear. Instead, she relied on encouraging statements from friends and relatives. “Everyone has told me that TAVI will make me feel like a new person”. When getting atrial fibrillation or other unexpected problems, they were disappointed. “Yes… I have said to anyone who asks that, if I had known it was so tough, I probably would not have agreed to do TAVI” 5.

### 3.4 Surprisingly simple rehabilitation

Far from all expressed disappointment. Instead, some of those who had been very worried before TAVI, were surprised that it was so easy and that they were grateful that this procedure was possible and had been offered to them.

#### 3.4.1 Perceiving TAVI easy to undergo

Our participants had experienced serious illnesses and surgeries before and, compared with their experiences, some thought that TAVI was easy to undergo. They expressed that they were lucky to have the opportunity to undergo TAVI instead of surgery; some because of less pain and time for recovery than from surgery and some because they would not probably have survived open surgery. “It was nothing, as I say when people ask, a piece of cake and it went just fine and I was almost cured before I came home” 12.

#### 3.4.2 Experiencing fast recovery

Several were surprised of how fast they recovered and that they could leave bed so quickly and walk around as compared with other patients who had done open heart surgery or with their own experiences from heart surgery. “The operation went so well, I never had problems afterwards, no pain either, more than when I got up the first day, but that was nothing. I recovered pretty fast. Hospital staff thought it was unusually fast…” 8.

### 3.5 Struggling with limitations

This category concerns limitations that remained 6 months after TAVI among some patients, mostly due to other diseases or old age.

#### 3.5.1 Being affected by other diseases

At follow‐up the participants’ heart problems were almost forgotten. Instead they reported many other ailments like having back pain, hip and knee problems or vision defects that were constantly present in everyday life. “It′s a pity that I have such a bad back, otherwise I would now have been as vigilant as anyone” 12. A man with chronic obstructive pulmonary disease said, “Of course, I still have difficulty breathing and it does not change, but I feel a huge difference compared to the past” 1. To make the most of the situation made it all easier. “I was so wise, so I asked for a walker when I left the hospital. It is an amazing thing. This was good because I sometimes become dizzy and then it's good support” 9.

#### 3.5.2 Being isolated and dependent

The patients in our study were old and afflicted by other diseases which had impaired their potential for a good social life and the TAVI treatment did not change that. They reported their situation as being lonely and isolated. It was described as difficult becoming dependent on others. “My daughter follows me everywhere. I cannot drive anymore because I have poor vision and hearing and I have diabetes” 2.

#### 3.5.3 Living only in the present

Several patients were tired and not motivated to do anything extra. They said that they were too old to think about the future or that the future was uncertain. “Well, when being old you don't think much ahead, you think more retrospectively about what has happened. You do not think so much about the future, you know which way it goes” 8. Despite that, they were mostly satisfied to live in the present and rejoiced of small happenings.

### 3.6 Returning to life

On the other hand patients said at the follow‐up, that they had got their lives back and received a new start in life. They had fewer problems breathing, had increased in weight and were in better condition, all of which made it more possible to stay independent, to take part in social activities and to plan for the future.

#### 3.6.1 Living in gratitude and joy

Happiness for having the opportunity to undergo TAVI was reported and satisfaction with their own decision, that they took the risk. They felt gratitude, both towards the doctors who recommended and treated them and the nursing team that supported them. “Yes, it feels like I've got my life back a little, I have been given another chance and I try to really take advantage of it and remember that I've actually have got another chance. I do things more spontaneously and I'm not waiting to do anything” 4. TAVI had influenced their psychological well‐being and attitude towards life; to feel joy just listening to the birds and observing nature, just being alive. “I want to go on living. I have three children, many grandchildren and great‐grandchildren that used to call me, they love me, so I want to stay alive a little longer” 3.

#### 3.6.2 Being independent

Their desire to stay independent was strong and was described as an important predictor of life quality. The participants were, despite illness, often socially active in different ways. Now they could take walks and do their own shopping, go to church and see friends and grandchildren more easily than before. Men described being able to drive as being very important and an expression of quality of life. “Now I feel that I have quality in life. I have taken out the car again and can drive it to the store, so it is no problem. I can cope by myself” 16. Some were living together with a partner that, due to high age, dementia or mental disorder, was dependent on them. It was described as a great relief to get well and be able to take care of their partner.

#### 3.6.3 Having hope for the future and zest for life

Feeling that there were improvements and fewer symptoms provided energy and hope for the future. Some said that they looked forward to the spring “it gives hope” and summer “to be able to mow the lawn” and they even had plans for building an extension of summerhouses. Having projects was described as giving meaning to life and it helped them forget problems. An example of having confidence in the future was expressed by a man who looked forward to elk hunting and had bought a puppy, knowing that it would take several years of training before he could hunt with the dog. Another was this statement from one of the women: “I remember that last year, when I took out the Christmas candle holders, I thought it was for the last time. Now, when we took them out again last week, I thought: my God, I believe I can use these several more years. I can't be sure but I imagine that I have some years left” 4.

## 4 DISCUSSION

The core concept in this study, “A journey of balancing between life‐struggle and hope,” illuminates patients’ experiences of the recovery process from TAVI. In almost all interviews there was a mix of those two dimensions. However, which one that was dominant varied depending on their experiences of illness and complications, the received support and their ability to cope with difficulties. In a study by Person and Rydén of effective coping in physical disability, they identified two bipolar core concepts: “Acknowledgements of reality vs. creation of hope” and “trust in oneself vs. trust in others” (Persson & Rydén, 2006). These concepts are similar to our findings and the coping strategies used among our participants throughout the whole TAVI trajectory.

The period before treatment was described as threatening but also hopeful. In critical stages in life, hope is considered to be an important coping strategy. Almost all patients were anxious about the rapid progression of their disease, an acknowledgement of reality. The creation of hope was made in connection with others. According to a study by Mollon, the attributes for feeling safe in hospitalization are trust, being cared for, presence and knowledge, leading to feelings of control, hope and calmness (Mollon, 2014). Schaufel, Nordrehaug, and Malterud (2011) made an interview study to explore how patients with life‐threatening diseases experienced hope when coping with mortality and other existential challenges. They found that hope could enhance and diminish existential distress and was described in the four themes: *Perceiving the realities of death—between overwhelming horror and peaceful acceptance*;* Adapting to a new phase in life—between reconciliation and uncertainty*;* Identity as seriously ill—between go‐ahead spirit and resignation;* and lastly, *The impact of relationship—between support and concern*. Their study reported that to be cared for by skilled personnel and hear the physicians say that they should together act and make it all turn out well, provided patients with hope (Schaufel et al., 2011). This is consistent with our findings where it was obvious that hope was created when patients felt respected and they participated in the treatment and when they trusted the doctors. Our participants generally stated that they felt involved in the decision‐making for TAVI. Shared decision‐making (SDM) is a way of implementing person‐centred care and incorporating the values and preferences of the patient in the decision‐making (Kremer, Ironson, Schneiderman, & Hautzinger, 2007). However, some of our participants did not want to be involved in decision‐making. Instead of “acknowledgements of reality” they “created hope” by listening to encouraging friends (cf. Persson & Rydén, 2006), a strategy that made them brave but probably less prepared for meeting problems during rehabilitation. Patients’ and doctors’ dialogues before high‐risk interventions have been studied by Schaufel and coworkers who found that when handling uncertainty, doctors communicated complex information about risks while patients required and trusted doctors’ advice (Schaufel, Nordrehaug, & Malterud, 2009).

According to findings of Berg et al. (2013), the rehabilitation after TAVI was described as demanding. In this study, a transapical approach was used and all patients were given full anaesthesia, which entailed intensive care afterwards. The necessity of early mobilization of elderly after surgery, to avoid complications and for the patients’ well‐being, is previously emphasized (Asher, 2004). This seems to be especially important in this group of older and fragile patients (Lauck, McGladrey, Lawlor, & Webb, 2016). Having a previous stroke was described as making the rehabilitation more difficult and help from physiotherapists and homecare aids was necessary. A study exploring hope from a nursing perspective, reports that goal‐oriented understanding of hope is particularly useful in the phase of rehabilitation (Tutton, Seers, & Langstaff, 2009). Despite being old and fragile, some participants in our study reported the opposite view and described TAVI as surprisingly easy. According to Snyder's theory, hope is defined as the competence to produce capable pathways to desired goals and motivation to use them. If barriers arise, such as complications or new diseases, high‐hope individuals are able to create new realistic goals, pathways and hopeful thinking (Snyder, 2000). This is in line with our findings where some of our male patients gave examples of creation of their own exercise programs and goals to recover and enhance hope and self‐trust when recovery was slow. For male patients with heart failure the importance of improving their physical capacity has also been highlighted by others (Mårtensson, Karlsson, & Fridlund, 1997). Congruent with findings by Schaufel and coworkers (Schaufel et al., 2011), many of our patients identified themselves as being people that did not give up and forced themselves to move forward. On the other hand, some faced hopelessness and at some point they had doubted that they would ever recover, which confirms the importance of inspiring hope in critical care (cf. Cutcliffe & Herth, 2002). Since TAVI patients have so many different problems, individual plans for rehabilitation are necessary, also a conclusion of others (Lauck, McGladrey, et al., 2016; Russo et al., 2014). Berg and Danielson emphasize the importance of caring relationship for patients in an exposed and vulnerable situation (Berg & Danielson, 2007). The ability to create continuity and trust by having contact with a specialist nurse is highlighted by Falk and coworkers (Falk, Wahn, & Lidell, 2007). Specialist nurses in the TAVI team could become a valuable resource in contact with patients, both during the preoperative investigations and planning, as well as after the procedure (cf. Lauck et al., 2013; Lauck, McGladrey et al., 2016).

The situation at 6 months follow‐up was described not only as struggling with limitations but as also a feeling of returning to life (cf. Berg et al., 2013). Other diseases are still present and, for some, had worsened and the struggle to manage life was still present. Despite that, most of our patients expressed gratitude and, since severe heart symptoms had decreased, living had become easier. This was also presented in self‐ratings (Olsson, Nilsson, Hornsten, & Naslund, 2017). Life had got a new meaning and they expressed happiness over simple things, a change in values also described by others (Andersson, Hallberg, & Edberg, 2008 Persson & Rydén, 2006; Rustoen, Howie, Eidsmo, & Moum, 2005; Schaufel et al., 2011). Men expressed that it was of great importance and a goal of continuing to drive. It was a way to stay independent but also to keep their masculine role intact, an important factor previously described in literature (Bosworth et al., 2004). Some had made up great plans for the future, while others were living only in the present and in the past. Charmaz has described this as a useful method to handle the uncertainty of future and thoughts of death for people suffering from long‐term illness (Charmaz, 1991). An important challenge for healthcare professionals is to help patients to preserve hopeful thinking that makes it possible to generate compensatory goals, such as trusting others and accepting assistance (cf. Gum & Snyder, 2002).

### 4.1 Study limitations

This study was conducted at a single centre that had little experience of TAVI treatment at the time of inclusion, something that may have influenced the result. Furthermore, all patients were treated transapical which may differ from experiences of patients treated transfemoral. The sample was not theoretical as recommended in Grounded theory since this is a part of a larger study that includes quantitative measures. There could be a risk for recall bias since interviews were performed 6 months after TAVI. Surprisingly, most participants gave a detailed and reflective picture of the recovery process.

## 5 CONCLUSIONS

This study demonstrates not only the stressful situation patients with severe aortic stenosis undergoing TAVI experience but also the changes the treatment causes in their view of life and hope for future. Other diseases are still present and, for some, have worsened and the struggle to manage life still remains. To have and preserve hope is essential for the patients’ well‐being, both before TAVI and during recovery process. It is an important that healthcare professionals not only support hopeful thinking but also take time to discuss and prepare patients before TAVI, talk about concerns and build a relationship to create the necessary confidence.

## 6 IMPLICATION FOR PRACTICE

Specialist nurses in the TAVI team could become a valuable resource in contact with patients, both during the preoperative investigations and planning, as well as after the procedure. Individual plans for rehabilitation, based on the needs of the patients and their resources, should preferably be designed by a team consisting of multiple professions together with the patient.

## CONFLICTS OF INTEREST

No conflict of interest has been declared by the authors.

## AUTHORS’ CONTRIBUTION

All authors have contributed substantially in design, interpretation of data and drafting the article. The first author made data collection and together with the last author analysed data.
